# Cancer clusters in the USA: What do the last twenty years of state and federal investigations tell us?

**DOI:** 10.3109/10408444.2012.675315

**Published:** 2012-04-21

**Authors:** Michael Goodman, Joshua S. Naiman, Dina Goodman, Judy S. LaKind

**Affiliations:** 1 Department of Epidemiology, Emory University, Rollins School of Public Health, Atlanta, Georgia; 2 LaKind Associates, LLC, Catonsville, MD, USA; 3 School of Arts and Sciences, University of Pennsylvania, Philadelphia, Pennsylvania, USA; 4 Emory College of Arts and Sciences, Atlanta, Georgia; 5 Department of Epidemiology and Public Health, University of Maryland School of Medicine, Baltimore, MD, USA; 6 Department of Pediatrics, Pennsylvania State University College of Medicine, Hershey, Pennsylvania

**Keywords:** Cancer cluster investigation, state health department, CDC, ATSDR, environmental agent, infectious agent

## Abstract

**Background:**

Cancer clusters garner considerable public and legislative attention, and there is often an expectation that cluster investigations in a community will reveal a causal link to an environmental exposure. At a 1989 national conference on disease clusters, it was reported that cluster studies conducted in the 1970s and 1980s rarely, if ever, produced important findings. We seek to answer the question: Have cancer cluster investigations conducted by US health agencies in the past 20 years improved our understanding of cancer etiology, or informed cancer prevention and control?

**Methods:**

We reviewed publicly available cancer cluster investigation reports since 1990, obtained from literature searches and by canvassing all 50 states and the District of Columbia. Investigations were categorized with respect to cancer type(s), hypothesized exposure, whether perceived clusters were confirmed (e.g. by elevated incidence), and conclusions about a link between cancer(s) of concern and hypothesized environmental exposure(s).

**Results:**

We reviewed 428 investigations evaluating 567 cancers of concern. An increase in incidence was confirmed for 72 (13%) cancer categories (including the category “all sites”). Three of those were linked (with variable degree of certainty) to hypothesized exposures, but only one investigation revealed a clear cause.

**Conclusions:**

It is fair to state that extensive efforts to find causes of community cancer clusters have not been successful. There are fundamental shortcomings to our current methods of investigating community cancer clusters. We recommend a multidisciplinary national dialogue on creative, innovative approaches to understanding when and why cancer and other chronic diseases cluster in space and time.

## Introduction

Examination of temporal-spatial clustering of disease in human populations occupies a prominent place in epidemiologic research. The history of epidemiology contains several examples of cluster investigations that led to landmark discoveries of disease etiology. Those include recognition of new infectious agents (Fraser etal., 1977; CDC, 2006), connections between nutritional deficiencies and human illness (Elmore & Feinstein, 1994; Scrimshaw, 2010), and identification of previously unknown carcinogens (Doll, 1975; Bosetti et al., 2003).

When considering disease clusters, it is essential to distinguish between investigations of clusters of infectious diseases and those of chronic, non-communicable conditions such as cancer. While etiologic investigations of infectious disease clusters have an impressive track record (Koo & Thacker, 2010), studies of cancer clusters are more complicated and less commonly lead to an identifiable cause (Thun & Sinks, 2004; Kingsley et al., 2007). Perhaps the most informative are studies of cancer clusters in which cases are linked by common occupation such as work with asbestos in a cluster of mesothelioma (Otte et al., 1990) or share an unusual risk factor such as prenatal exposure to diethylstilbestrol in a cluster of clear cell carcinoma of the vagina (Herbst et al., 1971).

By contrast, cluster investigations of cancers that appear to arise in a given geographic area or in a given community have rarely, if ever, resulted in important discoveries, atleastintheUSA(Rothman, 1990). Yetfew areas of epidemiologic research have captured so much public attention, providing material for best-selling books (e.g. A CivilAction), major motion pictures (e.g. Erin Brockovich) and articles in the popular media (e.g. Brodeur, 1992; Gawande, 1999). Moreover, it is the geographic cancer clusters that appear to attract sustained interest on the part of federal and state legislators as reflected in the recently introduced federal bill “Strengthening Protections for Children and Communities from Disease Clusters Act” (Boxer, 2011) and proposed state legislation (e.g. Maryland Senate Bill 574; Pennsylvania Legislation to Address Suspected Cancer or Disease Clusters).

The Centers for Disease Control and Prevention (CDC) National Center for Environmental Health (NCEH) defines a cancer cluster as “a greater-than-expected” number of cases that occurs “within a group of people in a geographic area over a defined period of time” (Kingsley et al., 2007). In any given year, there may be upwards of 1000-2000 inquiries about perceived disease clusters, most of them related to concerns about cancer (Thun & Sinks, 2004). When geographic clusters of cancer in a given community are suspected by the public and reported to an agency, a series of events is set in motion. Typically, the first response is a phone conversation in which the public health agency attempts to determine whether the inquiry is related to a commonly occurring cancer or multiple cancer types. In many cases, the cancer occurrences may be readily explained by, for example, demographics and in many if not most instances, this first communication is sufficient and no further activities are required (Drijver & Woudenberg, 1999). In some cases, however, the health agency is compelled to conduct a formal investigation that may involve an examination of cancer rates, an assessment of putative exposures, or both.

In 1989, a National Conference on Clustering of Health Events was convened to discuss empirical observations of disease clusters, advances in statistical methods for analysing cluster data, and risk perception and legal issues (the Conference proceedings were published in a special issue of the American lournal of Epidemiology; vol 132, Supplement, July 1990). In addition, the Conference speakers summarized the preceding 20 years of experience in conducting cluster investigations by health departments of several states (Fiore et al., 1990; Osborne et al., 1990) and by the CDC (Caldwell, 1990). The keynote speaker summarized the reasons why, in his view, studies of individual clusters do not advance our understanding of disease (Rothman, 1990): (i) individual disease clusters are too small to constitute useful epidemiologic research, (ii) reported clusters often involve vague or heterogeneous definitions of disease, (iii) the process of selecting the population of primary analytic interest is flawed by *a posteriori* reasoning, (iv) exposures are typically poorly defined or undefined and (v) the publicity generated by the cluster investigation can make unbiased data collection difficult or impossible.

Since the time of the 1989 Conference, states and the federal government have continued to investigate cancer clusters and new methodologies and protocols had been developed. However, to our knowledge, there has not been a systematic review of the community cluster investigations that have been conducted since the time of the Conference to ascertain whether these investigations contributed to our understanding of cancer etiology or advanced in anyway cancer prevention and control. The focus of this paper is on reports of investigations that examined geographic or community clusters of cancer, and specifically on those investigations that are initiated because of a perception of increased cancer rates in a community. While some investigations addressed other non-infectious chronic diseases such as multiple sclerosis, we focus on cancer cluster reports as these represent the preponderance of investigations conducted by state and federal agencies.

## Methods

### Identification of publicly available state and federal cancer cluster investigations

We first conducted general Internet and PubMed searches using the following key words in various combinations “cancer cluster” “disease cluster” “cluster investigation” Because cluster investigations only rarely lead to publications in scientific journals, we could not rely solely on searches of peer-reviewed literature. Thus, additional searches were conducted using the same search terms within websites for each individual state health department. As some cluster investigations are conducted by or in consultation with CDC or the Agency for Toxic Substances Disease Registry (ATSDR), we also examined electronic data sources maintained by these federal agencies. When relevant reports or publications were located, secondary references were reviewed and additional sources of information identified.

In addition to various searches of electronic sources, health departments in each of the 50 states and the District of Columbia were canvassed by telephone and/ or e-mail to determine whether all relevant cluster investigation reports had been located. If not, agency representatives were asked to send all remaining publicly available reports along with any other relevant information that they were at liberty to share.

### Inclusion/exclusion criteria for reports

We obtained information on several hundred reports from state agencies and/or their websites. Criteria for inclusion of a cluster investigation into the systematic review were (i) the reporting of a perceived geographic or community cancer cluster; (ii) a state or federal investigation that yielded a written publicly available report, a summary of an investigation, or a journal article and (iii) an investigation occurring between January 1990 and September 2011. Clusters were excluded from the review if (i) no formal state or federal investigation was conducted; (ii) the cluster involved an infectious disease or a non-cancer outcome; (iii) the cluster was occupational rather than residential; or (iv) the assessment of disease rates was initiated because of known concern about exposure (e.g. due to a nearby industrial site or a documented chemical spill), but without an *a priori* concern about elevated cancer rates or perception of a cluster.

Of the 2876 Health Assessments conducted by ATSDR and available on the Agency's website (http://www. atsdr.cdc.gov/hac/pha/index.asp), most were initiated because of concerns associated with known chemical exposures leading to an examination of cancer rates in the nearby population. We included only those reports that indicated that the ATSDR investigation was precipitated by a community concern regarding perceived increased cancer rates (i.e. presence of a cluster), as typically stated in the “Introduction” or in the “Community concerns” section, regardless of an *a priori* knowledge of specific chemical exposure; only those reports that contained a section on health outcomes data - indicating that a formal evaluation of cancer incidence (or mortality) rather than a risk assessment was conducted - were retained for further consideration. Some ambiguity existed in terms of distinguishing an investigation launched in response to a pre-existing concern about increased frequency of cancer in a given community from a methodologically similar, but conceptually different, investigation of cancer rates in an area with an environmental exposure problem (e.g. a chemical spill). For the purposes of this review, we are interested in the former category because it begins with a perceived aggregation of cancer cases “within a group of people in a geographic area over a defined period of time” which is the essence of a cancer cluster as defined by the CDC (Kingsley et al., 2007). If we could not discern whether or not the perceived cancer cluster preceded the known environmental issue, we retained the report in the review.

### Extraction of report information

Information extracted from each report included geographic location of the cluster, year of investigation, individual(s) reporting the perceived cluster, investigation agency, cancer site (or sites) of concern, evidence of increased frequency of the cancer(s) in question versus comparison rates (”confirmed” or “observed” cluster), environmental agents (if any) hypothesized to have been the cause of the cluster, and the final conclusions of the investigation. A cluster was considered confirmed if the report provided evidence of a statistically significant elevation in incidence (or mortality, if incidence data were not assessed) for the *a priori* stated cancer site(s) (e.g. all cancers, breast cancer, leukemia) and for the *a priori* identified subpopulations of concern (e.g. women, elderly, children). For example, if a concern was expressed about an increase in all cancers, but the investigation assessed multiple sites across multiple age-, gender- or race-specific population subgroups, and reported that only some of the rates were statistically significantly elevated without an overall increase in all cancers, the cluster was not considered confirmed. On the other hand, a perceived cluster of all cancers was considered confirmed if there was a statistically significant excess of the overall cancer incidence even if none of the individual site- or subpopulation-specific rates were significantly elevated. This approach was used for all states with the exception of Texas; in Texas, most community concerns were for “all cancers” but the investigators typically conducted the analyses by specific cancer site. When evaluating reports from Texas, perceived clusters were considered “confirmed” when the agency stated in the report that the cluster warranted further investigation or observation (even though in some cases further investigation was proposed without clear evidence of elevated disease rates).

If the *a priori* perceived cluster pertained to a specific site or category (e.g. brain tumors or leukemia), the cluster was considered confirmed in the presence of a documented statistically significant increase for that cancer site or category. When a particular report did not include a formal evaluation of cancer rates - e.g. because the analysis was limited to cases only, or because the report represented a case-control analysis of the association between the cancer(s) of concern and the exposure(s) of interest - a cluster was considered confirmed based on the information provided by the authors. For example, we included the post-1990 case-control analysis (Costas et al., 2002) of the childhood leukemia cluster in Woburn MA, which was investigated by the Massachusetts Department of Health prior to 1990 (Cutler et al., 1986). In this case, and in other similar instances, we assumed that a case-control study was initiated because the cluster was confirmed based on the earlier evidence.

When extracting information on suspected environmental risk factors, an attempt was made to be as specific as possible. For example, if a report mentioned that the main concern was related to volatile organic chemicals (VOCs) in the water supply and then listed the specific compounds, such as trichloroethylene (TCE) and tet-rachloroethylene (a.k.a. perchloroethylene [PCE]), we included TCE and PCE as the hypothesized exposures of interest. In many instances, however, the reports would simply mention “VOCs” or “chemicals from a nearby landfill” without identifying the specific compounds. Because the focus of this research was on cluster investigations originating with a concern about increased cancer rates, and not those that were the result of known chemical exposures, limited information was available on quantification of exposure (i.e. chemical concentrations in various media, estimates of intake).

Finally, we characterized each cluster investigation with respect to the presence or absence of an identifiable link between the cancer of concern and the hypothesized environmental exposure(s). In this categorization, we did not offer our own view on the presence or absence of the exposure-disease relation, but rather deferred to the authors’ conclusions.

## Results

### Description of cancer cluster reports

**Figure 1:**
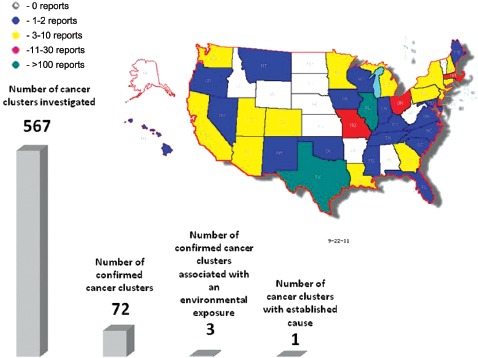
Numbers of publicly available cancer cluster investigation reports by state and comparison of numbers of investigated cancer clusters, confirmed cancer clusters (e.g. investigated clusters where number of cancer cases is greater than expected), clusters linked to an environmental exposure, and cancer clusters with an established cause. Although some of the cluster investigations may have been described in several reports, the numbers in this figure represent unique reported clusters. (Map generated from data in Table 1 using Map-Maker Utility, http://monarch.tamu.edu/∼maps2/us_12.htm)

We identified a total of 428 cluster investigations conducted in 38 different states (Figure 1, Table 1; see report references in Supplemental Material). In some cases, reports contained information on more than one investigation. In other cases, multiple reports addressing the same site were identified. As a result, the number of reports examined in this review was different from the total number of investigations. The remaining 12 states (AK, AL, AR, ID, KS, NV, ND, NE, SD, RI, WV, VT) and the District of Columbia did not have any relevant reports pertaining to cancer cluster investigations. The reasons for the variable number of reports differed by jurisdiction. For example, the representatives of the Alaska Cancer Registry indicated that they do not conduct cluster investigations because of low population density whereas in West Virginia and in Washington DC the clusters are apparently either not investigated, or if investigated, the records are not tracked. The typical practice of the Kansas Department of Health and Environment, the Nebraska Department of Health and Human Services, the North Dakota Cancer Registry and the Vermont Department of Health is not to publish cluster investigation reports. Instead, a response to an inquiry about a cancer cluster in those states is usually resolved either by phone, by mail or at public meetings.

**Table 1:** Summary of cancer cluster investigations by state.

State	Cancer sites of concern: number of perceived clusters^a^	Confirmed clusters cancer site: Number^b^	Hypothesized exposures: number of clusters	Link between cluster and hypothesized exposure
AL	NA	NA	NA	NA
AK	NA	NA	NA	NA
AR	NA	NA	NA	NA
AZ	Childhood leukemia:2, Brain:1	Childhood leukemia:1	Not listed:2, indoor air quality:1	No
CA	Multiple sites:1, all childhood cancers:2, childhood leukemia and lymphoma:1, childhood hepatoblastoma:1, pancreas:1, lung:1, breast:1, cervix:1, bone:1, paranasal:1, melanoma:1, acute lymphocytic leukemia:1, acute myelocytic leukemia:1, chronic myelocytic leukemia:1, leukemia:1, brain:1, ovarian:1	All childhood cancers:2, childhood leukemia and lymphoma:1, childhood hepatoblastoma:1, pancreatic cancer:1	Not listed:3, chromium:1, VOCs:1, dioxins:1, ionizing radiation, including radon:3, EMF:1, PCBs:1, pesticides:1, unspecified water contamination:1	No
CO	Multiple sites:2, brain:1	Brain cancer:1	Not specified:1, RF:1, PCE:1	No
CT	Multiple sites:2, childhood cancers:2, childhood leukemia:1, colon:1	None	Not listed:1, unspecified exposure from landfill and lagoon:1, VOCs:1	No
DE	Multiple sites:11, all childhood cancers:1, all female cancers:1, brain:4, breast:3, thyroid:1, Hodgkin's disease:1, pancreas:1, lung:2	All cancers:1, lung:1	Not listed:16, unspecified water contamination:4, exposures from coal burning facility:1, unspecified exposures from nearby plant:1	No
DC	NA	NA	NA	NA
FL	Multiple sites:1, brain:1	None	Unspecified exposure from nearby farms and citrus groves:1, aluminum:1, antimony:1, arsenic:1, boron:1, cadmium:1, chromium:1, fluoride:1, iron:1, lead:1, lithium:1, manganese:1, mercury:1, nickel:1, selenium:1, thallium:1, vanadium:1, sulfate:1, gross alpha:1, radium-226:1, radon-222:1	No
GA	Multiple sites:3, breast:1, glioblastoma multiforme:1, kidney and bladder:1	None	Not listed:1, unspecified water contamination:1, unspecified exposures from nearby carpet manufacturing facility:1, EMF:1, VOCs:2, PCBs:1, lead:2, cadmium:1, barium:1, radon:1, nitrates/nitrites:1	No
HI	Multiple sites:1, childhood leukemia:1	Childhood leukemia:1	EMF:1, pesticide exposures:1	No
ID	NA	NA	NA	NA
IL	Multiple sites:118, all childhood cancers:10, all brain cancers:5, brain glioblastomas:3, pediatric leukemia:3, breast:3, osteogenic sarcoma:2, brain medulloblastomas:1, multiple myeloma:1, nervous system:1, all adenocarcinomas:1, leukemia:1, lung adenocarcinoma:1, lung:1, non-Hodgkin's lymphoma:1	All cancers:7, breast:1, multiple myeloma:1, lung:1, all adenocarcinomas:1	Not listed:137, unspecified exposure from nearby landfill:1, asbestos exposure from unspecified source:1, low-level exposure from radioactive burial ground:1	No
IN	Breast:1, brain:2, pediatric brain/CNS:1, uterus:1, lung:1, leukemia:1	None	TCE:1, PCE:1, other VOCs:1, benzene:1, PCBs:1, lead:1, cadmium:1, barium:1, 1,2- dichloroethane:1, vinyl chloride:1	No
IA	Multiple sites:1, brain:1	Brain:1	Pesticides:1, EMF:1, radiation:1, solvents:1, animal viruses:1, N-nitroso-compounds:1, unspecified exposures from nuclear power plant:1	No
KS	NA	NA	NA	NA
KY	All or multiple sites:2	None	Technetium 99:1, dioxin:1, lead:1, benzene:1, 1,3-butadiene:1, 1,2- dichloroethane:1, ozone:1, chloroform:1, TCE:2, 1,1,2-trichloroethane:1, bromodichloromethane:1	No
LA	Multiple sites:3, neuroblastoma:1	Neuroblastoma:1	Not listed:2, dioxin:2, arsenic:1, barium:1, cadmium:1, lead:1, chromium:1, mercury:1, PAHs:1, VOCs:1	No
MD	Multiple sites:1	None	naturally occurring radioactivity in groundwater:1, potential air contaminants from point sources:1, other:1	No
ME	Multiple sites:1, brain:2, bone:1, lung:1	Brain:1	Unspecified exposures from pulp and paper mill and burning from municipal dump:1, chlorinated benzenes:1, unspecified exposures from nearby woolen mill complex:1	No
MA	Multiple sites:8, brain:2, brain/CNS:3, childhood cancers:3, Ewing family of tumors:1, breast:4, leukemia:4, melanoma:1, liver:1, lung:2, cervix:1, Hodgkin's disease:3, abdomen:1, colon:1, testes:1, non-Hodgkin's lymphoma:3, thyroid:2, prostate:1, stomach:1, childhood brain:1, childhood leukemia:1, kidney:2, bladder:1	Ewing family of tumors:1, breast:2, leukemia:2, melanoma:1, non-Hodgkin's lymphoma:1, prostate:1, brain:1, Hodgkin's disease:1, childhood leukemia:1	Not listed:7, unspecified chemicals:1, unspecified chemicals in drinking water:2, unspecified chemicals in swimming pools:1, proximity to landfill:3, diesel fuel release:1, PCBs:3, dioxins:2, radioactive magnesium thorium waste:1, RF:1, TCE:4, PCE:3, petroleum hydrocarbons:1, VOCs:4, metals:2, unspecified exposures from a power plant:1, pesticides:1, unspecified exposures from industrial sites:1, THMs:1, vinyl and asbestos cement in water pipes:1, nearby cranberry bog:1, unspecified exposures from chemicals from army lab:1, industrial site:1, arsenic:1, barium:1, cadmium:1, benzene:1, asbestos:1, chemicals from former dye manufacturer including benzidene, dianisidine, *o*-tolidine, napthylamine:1, unspecified environmental sites:1	One report (Costas et al., 2002) concluded “Results identified a non-significant association between potential for exposure to contaminated water during maternal pregnancy and leukemia diagnosis, (odds ratios 8.33, 95% CI 0.73–94.67). However, a significant doseresponse relationship (P ≶ 0.05) was identified for this exposure period. In contrast, the child's potential for exposure from birth to diagnosis showed no association with leukemia risk. Wide confidence intervals suggest cautious interpretation of association magnitudes.”
MI	Multiple sites:7	None	VOCs:3, unspecified exposures from nearby packaging plant:1, unspecified exposures from nearby explosives plant and barrel dump:1, hydrogen chloride:1, cement kiln dust:1, lead:2, benzene:1, carbon tetrachloride:1, formaldehyde:1, ammonia:1, phenol:1, ammonium sulfate:1, sulfuric acid:1, copper:1, manganese:1, phenylisocyanate:1, naphthalene:1, phenol:1, 1,2,4-trimethylbenzene:1, antimony:1, barium:1, cadmium:1, chromium:1, zinc compounds:1	No
MN	Multiple sites:2, breast:1, brain:1	All sites:1	Not listed:1, proximity to nuclear power plant:1, PAHs:1	No
MS	Multiple sites:1	None	Dioxin:1	No
MO	Multiple sites:9, brain:4, benign brain tumors:1, breast:1, liver:1, lung:3, prostate:2, oral cavity:1, lymphoma:2, non-Hodgkin's lymphoma:1, childhood testicular:1, melanoma:2, pituitary:1, testicular:1, colon:1, stomach:1, thyroid:1, childhood leukemia:1	None	Not listed:3, unspecified soil contamination:1, unspecified creek contamination:1, dioxin:3, unspecified landfill chemicals:1, EMF:1, charcoal kiln emissions:1, lead smelting chemicals:1, unspecified chemicals from beautification process:1, radiation fallout:1, unspecified chemicals from nearby oil refinery:1, pesticides:1, unspecified exposures from nearby explosives production plant:1	No
MT	Multiple sites:1, pancreas:1	None	diesel fuel:1, TCE:1, PCE:1	No
NE	NA	NA	NA	NA
NH	Multiple sites:4	None	Not listed:2, unspecified exposures from a coal tar waste deposit:1, arsenic:1, cadmium:1, chromium:1, nickel:1, lead:1, mercury:1, hydrogen chloride:1, dioxins and furans:1	No
NJ	Multiple sites:2, all childhood cancers:1, brain/CNS:1, astrocytoma:1, sympathetic nervous system tumors:1, neuroblastoma:1, Wilms' tumor:1, bone:1, soft tissue sarcomas:1, leukemia:1, acute lymphocytic leukemia:1, lymphoma and other reticuloendothelial neoplasms:1, Hodgkin's disease:1, non-Hodgkin's lymphoma:1	All childhood cancers:1, acute lymphocytic leukemia:1, brain and CNS:1, astrocytoma:1	Styrene-acylonitrile trimer:1, TCE:3, PCE:1, other VOCs:1, arsenic:1, lead:1	One report (NJDHSS, 2003b) concluded: “Several environmental factors of primary interest were found to be associated with leukemia in female children, specifically for the prenatal exposure time period. These associations were not found in male children.”
NM	Multiple sites:1, childhood cancers:1, thyroid:1, brain:1	Thyroid:1	radioactive air emissions and unspecified exposures from radioactive waste disposal:1	No
NV	Childhood acute lymphocytic leukemia:1, childhood acute myelocytic leukemia:1	Childhood leukemia:1	Unspecified exposures from naval air station:1, jet fuel:1, infectious agent carried by naval aviators:1	No
NY	Multiple sites:3, Hodgkin's disease:2, breast:1, childhood leukemia:1	Breast:1	Unspecified chemicals in drinking water and air:1, unspecified exposures from nearby landfill:1, EMF:1, pesticides:1, hazardous and municipal waste:1, VOCs:2, PCBs:2, PAHs:2, unspecified heavy metals:1, dibenzofurans:1, cadmium:1, chromium:1	No
NC	Multiple sites:1	None	Unspecified exposures from nearby landfill sites:1	No
ND	NA	NA	NA	NA
OH	Multiple sites:9, all childhood cancers:3, leukemia:2, thyroid:1, Hodgkin's disease:1, non- Hodgkin's lymphoma:1, multiple myeloma:1, brain/CNS:2	All childhood cancer:2, all sites:4	Not listed:5, unspecified exposures from local industries:2, unspecified exposures from nearby landfill:1, unspecified exposures from ordnance plant and engineer depot:1, mirex:1, methane:1, VOCs:1, TCE:1, unspecified metals from nearby landfill:1, environmental tobacco smoke:1, farm chemicals:1, infectious agents:1, molds:1, paint and paint thinners:1, solvents:1, lead:1, electrical transformer oils:1, construction materials:1, plastics in recycling:1, hazardous wastes:1, grease and glue:1, phenolics:1, benzene:1, naphthalene:2, benzo(a)pyrene:1, semi-VOCs:1, cyanide:1	No
OK	All or multiple sites:1	None	Unspecified exposures from Superfund site:1	No
OR	Brain:1, acute myelocytic leukemia:1	None	Pentachlorophenol:1, creosote:1, ammonia copper zinc arsenate:1, PAHs:1	No
PA	Multiple sites:3, osteosarcoma:1, colon:1, polycythemia vera:1, brain:1, breast:1, lung:1, stomach:1, leukemia:1	All sites:1, polycythemia vera:1	Unspecified exposures from former mill:1, unspecified exposures from acid mine tailings and waste-coal power plants:1, boron:1, trichloroethane:1, TCE:1, PCE:1, chloroform:1, hydrogen sulfide:1, sulfur dioxide:1, PAHs:1, aluminum:1, copper:1, lead:1, mercury:1, vanadium:1, zinc:1	No
RI	NA	NA	NA	NA
SC	Multiple sites:2, pleura:1	All sites:1, pleura:1	Asbestos:1, dioxins and VOCs from nearby incinerator:1	One report (Aldrich and Bolick, 1999) concluded “The level of pleural cancer deaths in this tri-county region of South Carolina is similar to that found in other parts of the country where asbestos related industries have been concentrated...”
SD	NA	NA	NA	NA
TN	Multiple sites:1	None	Radiation	No
TX^c^	Multiple sites:96, childhood cancer:6, colon and rectum:2, pseudomyxoma peritonei:1, lung:4, liver:4, thyroid:2, multiple myeloma:1, brain:4, brain/CNS:1, glioblastoma:1, childhood brain cancer:1, kidney:2, breast:2, leukemia:1, cervix:1, leukemia:5, childhood leukemia:3, multiple myeloma:1, bladder:1, all lymphomas:1, non-Hodgkin's lymphoma:2, Hodgkin's disease:2, soft tissue sarcoma:1	Multiple sites:4, childhood cancer:1, brain/CNS:1, stomach:1, multiple myeloma:1, liver and intrahepatic bile duct:2, Hodgkin's disease:2, lung:1, sarcoma:1, breast:1	Not listed:44, unspecified chemicals in drinking water:7, unspecified exposures from golf course:2, landfill:5, nearby industry:4, asphalt plant:1, poultry farms:1, dump sites:1, creosote plants:1, fertilizer plant:1, petroleum distribution facility:1, petrochemical plants:2, lake:1, closed air base:1, Superfund sites:1, nuclear waste dump:1, refineries:2, army munitions plant:1, power plant:1, oil and gas wells:1, army training site:1, pipelines:1, arsenic:10, PCE:1, TCE:5, dichloroethene:3, vinyl chloride:1, THMs:4, benzene:9, 1,3-butadiene:3, radiation:3, EMF:1, nitrates:2, 1,2-dichloroethane:1, manganese:3, PCBs:2, DDT:2, toxaphene:2, chromium:5, cadmium:3, copper:2, silver:2, mercury:2, selenium:2, aluminum:2, barium:2, beryllium:2, vanadium:3, lead:3, antimony:2, nickel:3, zinc:2, cobalt:1, ammonia:1, sulfur dioxide:1, aluminum oxide:1, ethylene oxide:1, propylene oxide:1, vinyl acetate:1, acrylonitrile:1, asbestos:2, PAHs:2, pentachlorophenol:1, dioxins/furans:1, VOCs:1, diesel exhaust:1, disinfection byproducts:1, occupational lignite exposure:1, pesticides:1	No
UT	Multiple sites:6, brain:1, lung:1, soft tissue:1, lymphocytic leukemia:1, non-Hodgkin's lymphoma:1	Brain:1, lung:1	Proximity to vanadium/uranium processing mill:1, dioxin:1, unspecified chemicals in groundwater from air force base:1, TCE:1, PCE:1, carbon tetrachloride:1, perchlorate:1, proximity to gravel pit, asphalt plant, storage facility:1, nitrates in ground water:1	No
VT	NA	NA	NA	NA
VA	Multiple sites:1	None	Unspecified chemicals from nearby building material plant:1	No
WA	Multiple sites:2, childhood cancers:1, acute lymphocytic leukemia	Acute lymphocytic leukemia:1	Ethylene dibromide:1, 1,2-dichloropropane:1, PAHs, mercury and unspecified chemicals from nearby former missile launch site:1, VOCs:1, semivolatile compounds:1, heavy metals:1, PCBs:1, pesticides:1, PAHs:1	No
WV	NA	NA	NA	NA
WI	Multiple sites:1, chronic lymphocytic leukemia:1	Chronic lymphocytic leukemia:1	Pesticides:1, VOCs:1, chloroform:1, carbon tetrachloride:1	No
WY	NA	NA	NA	NA

a May not add to total as some clusters may include several cancer categories. “Multiple sites” indicates that the community concern did not center on one or more specific cancer sites, but rather noted a general increase in cancers. Similarly, “all childhood cancers” indicated a community concern for childhood cancer generally, not for a specific type of childhood cancer.

b Defined as evidence of statistically significant elevation in cancer incidence consistent across population groups for the a priori stated cancer site(s).

c Texas: most community concerns were for “all cancers” but the investigation typically subdivided the cancers by type. For Texas, we include those cluster investigations in which Texas DOH recommended further investigation as “confirmed clusters”; further investigation may be proposed without evidence of a confirmed cluster.

CNS, central nervous system; DDT, dichlorodiphenyltrichloroethane; EMF, electromagnetic field; NA, not applicable; RF, radiofrequency; PAHs, polyaromatic hydrocarbons; PCBs, polychlorinated biphenyls; PCE, perchloroethylene; TCE, trichloroethylene; THMs, trihalomethanes; VOCs, volatile organic chemicals.

As shown in Figure 1, the number of cluster investigations available from each of the 38 states that had at least one report ranged widely. The greatest numbers of investigations were from Illinois *[N=* 139) and Texas *[N=* 119), while four other states - Massachusetts, Delaware, Missouri and Ohio - had 28, 22, 17 and 13 investigations, respectively. All other states had fewer than 10 investigations.

Most investigation reports provided by the states involved a comparison of observed and expected rates based on surveillance data and/or a *de novo* epide-miologic case-control study. However, some states (e.g. Missouri) limited their initial assessment to an examination of case series and would stop their investigation after determining that the reported cancer cases in a perceived cluster were too dissimilar or too spread over time and/ or space to warrant further study.

With respect to the main sources of cluster investigations included in this review, 367 reports were obtained from state health departments, 56 were derived exclusively from ATSDR and only 5 reports of investigations (2 from Hawaii and 1 each from Nevada, Pennsylvania and Massachusetts) were obtained from the peer-reviewed literature. Some of the 367 reports provided by the states were supplemented by additional information obtained from ATSDR and/or from our search of the peer-reviewed literature.

### Summary of findings

The 428 cluster investigations summarized in Table 1 assessed community concerns pertaining to 567 cancer sites or categories (including the category “all cancers”). It is clear from Table 1 that some states (e.g. Illinois and Texas) conducted multiple investigations of perceived clusters for a wide array of cancers. The two largest states in terms of population size (California and New York) contributed a relatively modest number of publicly available investigations (8 and 5, respectively). We were informed that New York conducted numerous investigations between 1990 and 2010, but most of these were not included in Table 1 as they are not public documents. California was the only state for which we were unable to contact a representative; however, the California Department of Health Services allows access to an extensive on-line repository of investigations, including investigations of cancer clusters, all of which were evaluated in our review.

Table 1 also shows that the public concerns regarding environmental exposures included a broad array of chemical and industry categories, but there appeared to be no discernable pattern. Of the 567 cancer sites or categories, a perceived increase in incidence was confirmed for 72 (13%) cancer types. Three reports (0.7% of 428 total investigations or 0.5% of 567 total cancer types assessed) indicated that at least some evidence was found of an association between the cancer(s) of concern and hypothesized exposures, although the level of certainty of these findings differed.

The first report with evidence of an association between the cancer of concern and hypothesized exposure was an investigation of a pleural cancer cluster in the Charleston area of South Carolina (Aldrich & Bolick, 1999) which revealed a pronounced excess risk for pleural cancer in a single ZIP code. An expanded investigation of pleural cancer incidence across the surrounding tri-county area found a statistically significant fourfold increase in rate for pleural cancer compared to that expected based on a statewide estimate (Aldrich & Bolick, 1999). Mapping revealed close aggregation of cases in the tri-county region that was in sharp contrast to the widely dispersed patterns in the remainder of the state. In addition, it became clear that the pattern of excess in pleural cancers had persisted in the tri-county area for over 20 years. A systematic review was made of the occupations of the 19 pleural cancer cases comprising the tri-county cluster. Twelve of the 19 cases were determined to have worked at the Charleston naval shipyard (Aldrich & Bolick, 1999), a finding that is consistent with the previously well-established increase in pleural cancer risk among asbestos-exposed shipyard workers.

The second report, a pediatric leukemia cluster in Woburn, MA, has been evaluated in several scientific publications and reports, and has received considerable attention from the media and entertainment industry. The cluster was confirmed in earlier investigations (e.g. Cutler et al., 1986), which reported that leukemia incidence in Woburn was clearly increased in boys (but not in girls), and also reported no significant differences in medical or exposure characteristics between the leukemia cases and those in the two matched control groups. The data were re-analysed by Costas et al. (2002) who examined the association between leukemia and exposure to water from two contaminated wells (G and H) over four different time periods: from 2 years before conception to diagnosis, during the 2 years before conception, during pregnancy and from birth to diagnosis. Although none of the odds ratios was statistically significant, there was evidence that in one of the four time intervals examined (during pregnancy) there was a significant dose-response relation (p for trend <0.05) between the levels of exposure to water from the two contaminated wells (defined as “never” “least” and “most”) and leukemia.

The third report described a cluster of pediatric cancers in Dover Township, New lersey and in particular in its Toms River section. Significant elevations were found among Toms River girls (but not boys) under 5 years of age specifically for acute lymphocytic leukemia and cancers of the brain and central nervous system (CNS) (New lersey Department of Health and Senior Services [NIDHSS], 2003a). The environmental exposures hypothesized to be responsible for this cluster included water and air contamination from the nearby industrial sites. The analyses of the relation between leukemia for different age groups produced multiple odds ratios; some were significantly elevated, others were significantly decreased and most were near unity. After summarizing their findings, the authors concluded that several environmental factors of primary interest were found to be associated with leukemia in female children, specifically for the prenatal exposure time period. These associations were not found in male children (NIDHSS, 2003b).

### Most commonly investigated cancer sites and hypothesized exposures

The most common cancer sites in the cluster investigations were leukemia/myeloma and breast and brain cancers. The only category reported with greater frequency was the generic “all cancers” which accounted for 281 of the perceived clusters. As shown in Table 2, in 24 (50%) of the 48 brain cancer clusters investigated, the reports did not mention any hypothesized causal factors. A wide array of chemicals/exposures - either specific (e.g. cadmium, TCE) or vaguely defined (e.g. proximity to a landfill), and often several listed together - were hypothesized as being linked to brain cancer, but none of these hypotheses found support for a variety of reasons, including (i) the perceived increase in brain cancer incidence was not confirmed, (ii) there were no exposure data, (iii) available exposure information or mapping information did not suggest an association or (iv) investigation of cause was outside the purview of the investigating agency. As with brain cancer, for a large percentage of perceived breast cancer clusters and leukemia/myeloma clusters (12 of 20 [60%] and 16 of 39 [41%], respectively), no hypothetical causal factors were listed (Table 2). For the remainder of the breast and leukemia/myeloma clusters, there was again a wide range of environmental factors indicated as the exposure of concern. Overall, brain cancers, breast cancers and leu-kemias/myeloma were linked to 35, 25 and 43 different exposure categories, respectively.

**Table 2:** Summary of exposures hypothesized to be linked to three most commonly perceived cancer clusters.

Brain cancer clusters (*N* = 48)^a^		Breast cancer clusters (*N* = 20)^*^		Leukemia and myeloma clusters (*N* = 39)^a^	
		
Exposures of concern	*N*	Exposures of concern	*N*	Exposures of concern	*N*
Not listed	24	Not listed	12	Not listed	16
Radiation	4	Lead	1	VOCs	4
Dioxin	2	TCE	4	TCE	4
CO_2_	1	VOCs	1	PCE	3
VOCs	1	TCA	1	EMF	1
Pesticides	2	Benzene	1	Radiation	1
EMF	1	Trihalomethanes	1	Lead	2
Solvents	1	Radon	1	Radon	1
Animal viruses	1	PCE	3	PCBs	2
N-Nitroso compounds 1,2-Dichloroethane	1 1	PCBs Barium	1 1	Styrene-acrylonitrile trimer Trihalomethanes	1 1
Vinyl chloride Styrene-acrylonitrile trimer Chlorinated benzenes TCE	1 1 1 2	Cadmium EMF Vinyl/asbestos cement PAHs	1 2 1 1	Benzene PAHs Pentachlorophenol Cadmium	1 3 1 1
PCE	3	Chloroform	1	Barium	1
Pentachlorophenol Creosote	1 1	Metals Pesticides	1 2	Creosote Ammonia Cu Zn arsenate	1 1
Ammonia Cu Zn arsenate	1	*Unspecified exposure from*:		Aluminum	1
Boron	1	Contaminated drinking water	2	Arsenic	1
Benzene	1	Air pollution	1	Chromium	2
PCBs	1	Power plant	1	Copper	1
PAHs	1	Nuclear generating plant	1	Mercury	1
Lead	1	Hazardous/municipal waste	1	Vanadium	1
Cadmium	1	Other sites	1	Zinc	1
Barium	1	Transformers and power lines	1	Dioxin	1
*Unspecified exposures from*:		Indoor and ambient air pollution	1	Ethylene dibromide	1
Carpet industry	1			1,2-Dichloropropane	1
Chemical manufacturer	2			Pesticides	3
Radio towers	1			Metals	1
Landfill	1			Vinyl/asbestos cement	1
Industrial site	1			60 Hz magnetic field	1
Pulp and paper mill	1			Jet fuel	1
Burn/municipal dump	1			Infectious agents	1
Refinery	1			*Unspecified exposures from*:	
Groves and farms	1			Contaminated drinking water	1
Transformers and power lines	1			Power plant	1
				Other sites	1
				Explosives production plant	1
				Naval air station	1
				Landfill	1
				Superfund site	1
				Army ammunition plant	1
				Transformers and power lines	1
				Ordnance plant and engineer depot	1

a Several cluster investigations listed more than one hypothesized exposure.

EMF, electromagnetic field; PAHs, polyaromatic hydrocarbons; PCBs, polychlorinated biphenyls; PCE, perchloroethylene; TCA, trichloroethane; TCE, trichloroethylene; VOCs, volatile organic chemicals.

The three most commonly identified chemicals of concern to communities inquiring about perceived cancer clusters were TCE (20 clusters), benzene (16 clusters) and dioxin (15 clusters) (Table 3). A wide array of cancer types was hypothesized to be linked to each of these three chemicals, with several of the cluster investigations evaluating more than one cancer category. The most common cluster category was “all cancers” which was identified (often along with other more specific categories) as an *a priori* concern in 75%, 94% and 73% of TCE-, benzene- and dioxin-related clusters, respectively. With respect to more specific cancer categories (not counting all childhood cancers), communities expressed concern about 21 different organ sites and TCE exposure, 7 different organ sites and benzene, and 7 organ sites and dioxin.

**Table 3:** Summary of cancers hypothesized to be linked to three most common community-identified chemicals.

Trichloroethylene (TCE) (*N* = 20)^a^		Benzene (*N* = 16)^a^		Dioxin (*N* = 15)^a^	
		
Cancer sites	*N*	Cancer sites	*N*	Cancer sites	*N*
All cancers	15	All cancers	15	All cancers	11
Breast	4	Brain	2	Lung	2
Leukemia	4	Lung	2	Brain	2
Brain/CNS	2	Breast	1	Pancreas	1
Lung	2	Uterus	1	Neuroblastoma	1
Uterus	1	Leukemia	1	Melanoma	1
Abdomen	1	Bone	1	Colon	1
Colon	1	Childhood - all	1	Stomach	1
Testes	1				
Kidney	1				
Pancreas	1				
Childhood - all	1				
Astrocytoma	1				
Sympathetic nervous system	1				
Neuroblastoma	1				
Wilm's tumor	1				
Bone	1				
Soft tissue sarcoma	1				
Lymphoma	1				
Hodgkin's disease	1				
Non-Hodgkin's lymphoma	1				
Stomach	1				

a Several cluster investigations examined more than one cancer category.

## Discussion

To our knowledge, this paper represents the first attempt to assemble and systematically review all publicly available state and federal cancer cluster investigation reports generated over the past two decades. This type of review permits an assessment of progress in the field of cancer cluster investigations - specifically, whether the effort expended by state and federal health agencies in the USA over the past 20 years has contributed to our understanding of cancer etiology or informed cancer prevention and control.

While cluster investigations serve many purposes, there is an expectation on the part of the public that cancer cluster investigations will reveal an environmental exposure that is causally related to the perceived or observed increase in number of cancers in a community (Congressional Staff Report, 2009). Currently proposed federal legislation in the USA would provide additional resources to the current effort to understand causes of cancer clusters (Boxer, 2011). At issue is whether the public, legislators and others should expect improvements in identifying causes of cancer by conducting cluster assessments and/or allocating additional resources to these types of investigations, and if not, whether other benefits of conducting cancer cluster investigations are commensurate with the resources expended. We explore this issue by discussing the following questions: (1) What does the evidence from cancer cluster investigations tell us? (2) Are there other avenues that might prove fruitful? (3) Are cancer cluster investigations worth pursuing? (4) Is there reason to believe that additional state and federal resources allocated towards cancer cluster investigations will advance our understanding of cancer etiology?

### What does the evidence from cancer cluster investigations tell us?

Reports of cancer clusters in the scientific and medical literature date back to the turn of the 20th century (Boyle et al., 1996). Since that time, thousands of cancer clusters have been investigated, but the goals from agency to agency have not necessarily been uniform. For example, some state health agency representatives informed us that the main goal of their investigations was to educate the public regarding cancer screening and cancer prevention methods such as cessation of cigarette smoking, reducing sun exposure and improving diet. Evaluation of the efficacy of public education by conducting cancer cluster investigations is outside the scope of the review. However, if the goal is to enhance our understanding of disease etiology and ultimately reduce the number of cancers, then based on the results of this review it is clear that this goal has not been met and appears unlikely to be met in the future.

In conducting the current review, we have assembled and reviewed reports pertaining to over 400 cancer cluster investigations and in only one of them was the cause identified with certainty (Figure 1). In a cluster of pleural cancer (presumably mesothelioma) in coastal South Carolina, there was clear evidence that the excess in cases was attributable to history of work at a nearby shipyard. This investigation did not provide novel information regarding etiology of mesothelioma because the increase in rates of this malignancy has already been well-documented in shipyard areas onboth coasts (Jemal et al., 2000; Teta et al., 2008). The other two investigations that reported an association between leukemia clusters and environmental exposures were less conclusive; both found an association in only one population subgroup (boys, but not girls, in Massachusetts and girls, but not boys, in New Jersey), and both appropriately recommended caution due to statistical uncertainty (Costas et al., 2002; NJDHSS, 2003b). More importantly, unlike the association between mesothelioma and asbestos reported from South Carolina, the findings from New Jersey and Massachusetts have not been confirmed in other settings.

The results of this systematic review point to the ineffectiveness of geographic cluster investigations as a means of discovering causes of cancer and are in accordance with earlier reports. For example, a review of 108 community cancer clusters investigated by the CDC from 1961 to 1982 found that a well-established cause was not identified for any of these clusters (Caldwell, 1990). In 1990, Minnesota reported results from over 500 investigations of clusters, 6 of which were full-scale investigations; in only one case, in an occupational setting, was there an important public health outcome concerning the cancer in question (CDC, 1990).

### Are there other avenues that might prove fruitful?

In this review, we found that the causal hypotheses aiming to explain cancer cluster etiology overwhelmingly implicated some form of environmental contamination from nearby industrial sources. By contrast, only three investigations (one of a leukemia cluster, one of all childhood cancers, and one of a brain cancer cluster) hypothesized that the disease etiology may be explained by an infectious agent. The apparent lack of focus on a potential infectious etiology is somewhat surprising, especially for leukemia, considering that leukemia has shown a propensity towards clustering in space and time, a feature most commonly associated with infectious diseases (McNallyetal., 2009).

In 1988, Kinlen observed that leukemia clusters tend to occur in isolated geographic areas that undergo a sudden influx of population, an observation that indirectly supports possible infectious etiology (Kinlen, 1988). Since then, Kinlen's Population Mixing Hypothesis has been supported by several similar investigations in the United Kingdom (Kinlen & Balkwill, 2001), Sweden (Kinlen et al., 2002), the United States (Wartenberg et al., 2004), Hong Kong (Alexander et al., 1997), Canada (Koushik et al., 2001), and Croatia (Labar et al., 2004). Not all studies were able to support the Population Mixing Hypothesis (Law et al., 2003; Dockerty, 2009); however, given the lack of success in identifying other environmental causes of clusters to date, consideration of infectious agents for leukemia, and possibly for certain other cancer types, is warranted. In fact, a recent study of the Fallon, Nevada cluster of childhood leukemia concluded that the space-time pattern of disease was suggestive of an infectious etiology (Francis et al., 2011) and the authors note that “specific infections have not been adequately addressed in any leukemia cluster investigation”

Although much of the public concern regarding cancer clusters is focused on exposure to industrial pollutants, other modifiable factors such as lifestyle characteristics are generally considered the major contributors to cancer etiology (Parkin etal., 2011).Thus, acompelling argument can be made that investigations of cancer clusters should take into consideration environmental risk factors in a much broader sense (i.e. those that include biological, socioeconomic and lifestyle-related factors). The evidence that lifestyle and socioeconomic factors play a role in the spatial distribution of cancer incidence is ample. For example, data from the US Surveillance Epidemiology and End Results program show that counties with lower poverty, higher education, higher income, and lower unemployment have higher age-adjusted rates formelanoma (Singh et al., 2011). By contrast, areas with higher levels of poverty and less educated population are consistently characterized by higher incidence of lung and cervical cancer (Shack et al., 2008; Clegg et al., 2009).

The observed geographic disparities in cancer incidence are likely attributable to differences in risk factors such as cigarette smoking, poor diet, physical inactivity, obesity, sexual practices, and health care seeking behaviors, most notably utilization of screening. Klassen et al., 2005 illustrated the role of socioeconomic factors by evaluating the spatial distribution of prostate cancer cases reported to the Maryland Cancer Registry. Initial cluster detection analyses, prior to adjustment, indicated that there were four statistically significant clusters of high and low prostate cancer rates. After adjustment for individual case attributes, including age, race and year of diagnosis, patterns of clusters changed. Additional adjustment for census block group and county-level socioeconomic measures further changed the cluster patterns.

### Are cancer cluster investigations worth pursuing?

According to the CDC guidelines for investigating clusters of health events “In many reports of cluster investigations, a geographic or temporal excess in the number of cases cannot be demonstrated... When an excess is confirmed, the likelihood of establishing a definitive cause-and-effect relationship between the health event and an exposure is slight” Based on this statement made over 20 years ago and on the more recent information summarized in this paper, it is reasonable to ask why health agencies continue to investigate clusters in general, and cancer clusters, in particular.

We noted above that some health departments view cluster investigations as a means to educate the public regarding cancer prevention and screening. In addition, cluster investigations may be warranted when the community believes there is a legitimate concern because those concerns may grow out of proportion if no official response is forthcoming (Bender, 1987). The CDC has observed that “the perception of a cluster in a community may be as important as, or more important than, an actual cluster” However, not all attempts to communicate the results of cluster investigations may be successful particularly if community representatives are not satisfied with epidemiologic or statistical arguments that do not support their concerns (Bender et al., 1995; Winn, 2005).

### Is there reason to believe that additional state and federal resources towards cancer cluster investigations will advance our understanding of cancer etiology?

In recent years, there has been a renewed interest in legislation related to disease clusters. States such as New York (NY, 2009), Pennsylvania (Yudichak, 2011), and Maryland (MD, 2011) have considered or proposed legislation related to facilitating cluster investigations. At the federal level, a bill (S76) titled “Strengthening Protections for Children and Communities from Disease Clusters Act” also referred to as Trevor's Law, has been introduced, which would direct the Environmental Protection Agency to investigate and address cancer and disease clusters (Boxer, 2011). The target populations of the Act are “pregnant women, infants, children, and other individuals who have been, are, or could be harmed by, and become part of, a disease cluster” The Act's goals include enhancement of “Federal resources, expertise, outreach, transparency and accountability in responding to public and State and local government inquiries about the potential causes of a disease cluster”; strengthening “Federal analytical capacity and coordination... in the investigation of the potential causes of disease clusters”; development of multidisciplinary teams that would use a “systematic, integrated approach to investigate and help address the potential causes of disease clusters that State and local officials cannot address or need assistance in addressing”; and helping “facilitate the rapid investigation of potential disease clusters and actions to address the potential causes of disease clusters”

The recent increase in state and federal legislative activity pertaining to cancer clusters with specific focus on environmental pollutants is difficult to understand in view of the lack of progress in this area even after four decades of study. Public health policy should be determined on the basis of our best understanding of the scientific data. This review as well as earlier synopses of cancer cluster investigations suggest that unsuccessful outcomes for cluster investigations are not due to lack of resources (in this study, states with resources to conduct more in-depth investigations were no more likely to draw definitive conclusions than those that produced only a few reports). Rather, a compelling argument can be made that past and current approaches towards cancer cluster investigations are flawed. As was noted in the editorial accompanying the proceedings from the 1989 National Conference on Clustering of Health Events, the unique aspect of a cancer cluster investigation is the reactive nature of the study, often from “data toward a hypothesis, in stark contrast to the scientific method of developing a hypothesis first and then gathering data to confirm or deny it” (Anonymous, 1990).

The methodological problems pertaining to investigations of community cancer clusters fall into several categories. First, a false perception of a cluster may result from failure to consider changes in population size over time and inability to account for migration in and out of the community (Aldrich & Sinks, 2002). A separate problem is boundary shrinkage, defined as bias in defining the boundary of a cluster leading to the overestimation of the disease rate (Olsen et al., 1996). As noted by Bender et al. (1995) the comparison of observed and expected numbers (as in calculating the standardized incidence ratios) when applied to populations known *a priori* to be unusual invalidates the laws of probability and renders resultant estimates meaningless. Bender et al. (1995) likened this approach to selecting one's lottery numbers after the lottery is completed, whereas Rothman invokes the proverbial Texas sharpshooter “who first fires his bullet and only then draws the target” (Rothman, 1990). Another problem with investigating geographic clusters is the tendency to conduct multiple analyses at the same time. With this approach some geographic units (e.g. counties or census tracts) and some subpopulations (e.g. sex-, age- or race-specific groups) may indeed show higher frequency of disease compared to their neighbors or the larger surrounding area. The likelihood of finding a statistically significant result increases further when the analyses simultaneously examine multiple cancer sites. For example, an analysis of cancer incidence by county in Minnesota identified 100,000 “clusters” for 85 cancers; 10,000 of these exceeded the statewide rate at least twofold, and 1500 were statistically significant. A similar analysis for towns, neighborhoods, or school districts would likely produce an infinite number of “clusters” (Williams, 1998). It is logical to conclude that in the absence of innovative approaches for examining cancer clusters, additional resources in and of themselves are unlikely to improve the current situation.

Additional problems that limit our ability to investigate cancer clusters include absence of data pertaining to relevant current or past environmental exposures, low statistical power of most analyses stemming from small population sizes (Thun & Sinks, 2004), the need to consider perception issues in situations where cluster investigations are highly publicized (Rothman, 1990; Blake, 1995), and vague definitions of disease (e.g. “brain tumors” or “leukemia”) that often include dissimilar conditions characterized by different pathogenesis and histologic features and, likely, different etiology. For conditions other than cancer, cluster investigations are further complicated by the absence of population-based disease registries capable of providing data on background rates (Rothenberg et al., 1990).

Our conversations with state representatives indicate that they are fully cognizant of the difficulties facing cluster investigations. In reviewing cancer cluster investigation practices, Kingsley et al. (2007) reported that while state protocols “were continuing to evolve” most states and territories take “a systematic approach” which includes the use of “standardized forms to facilitate information gathering, triage of incoming inquiries, and general adherence to the framework suggested by the 1990 CDC guidelines” (CDC, 1990). Moreover, according to Kingsley and colleagues “all states and territories were well aware of the inherent complexities in cancer cluster investigations, including data quality, migration, latency, small numbers, and political issues”.

In summary, we reviewed over 400 cluster investigations pertaining to hundreds of cancer categories conducted during the past two decades and found that only 72 out of 567 of those investigations confirmed a statistically significant increase in cancer rate. Only 3 investigations reported an identifiable link with an environmental cause, and of those, only 1 could be described unequivocally as an etiologic cluster.

Our review had several limitations. First, while we were able to access hundreds of reports for this research, our review likely did not capture the entirety of cluster investigations over the previous two decades. For example, while we canvassed every state health agency and requested all available reports, several states could not make reports available to us either because they did not have a mechanism for sharing them (e.g. some reports may not be readily accessible) or could not share them (e.g. some reports contain private information). In addition, several states investigated clusters, but did not produce formal reports. Oftentimes, results of investigations were simply shared by telephone with concerned parties. However, based on our conversations with state health departments, it appears unlikely that important investigations yielding information on cancer cluster etiology were missed. For example, a representative from one health department that could not provide specific reports indicated that they conducted over 300 investigations during the previous few decades and had not identified a causal factor in any of these. The second limitation relates to our inclusion/exclusion criteria for ATSDR Public Health Assessments. In this review, we sought to limit our examination of cancer cluster investigations to those that arose from community concerns regarding perceived increases in cancer rates in a geographic area. In some cases, the rationale for the investigation was unclear. In these cases, we erred on the side of inclusion rather than exclusion. In preparation of this review, we planned to determine whether highly publicized cluster investigations led to a change in the numbers of reports. However, in many states there were too few investigations in general for a trend to be noted. In others, the numbers depended in part on the budget.

Finally, we recognize that we did not include certain ongoing, high visibility cluster investigations such as the cluster of cancers in Camp Lejeune, North Carolina. These were not reviewed because completed investigation reports were not available. We did not see evidence of a sufficiently large number of these current investigations such that our overall conclusions would change.

## Conclusions

Twenty-two years after the National Conference on Clustering of Health Events that reported few, if any, successful investigations in the preceding two decades, it is fair to state that an extensive nation-wide effort to find environmental causes of community cancer clusters has not been successful. This is by no means the fault of the researchers and state or federal agencies conducting these investigations, but rather a reflection of the fundamental methodological problems pertaining to this type of activity. The reasons for disappointing results have been described in this review and elsewhere (Aldrich & Sinks, 2002; Thun & Sinks, 2004; Kingsley et al., 2007) and in fact were discussed extensively at the 1989 Conference.

At a time, when cancer research funding is scarce, it is time to pose the following questions: Given the outcomes of community cancer clusters investigations over the past 40 years, is it appropriate that we devote more resources to staying the same path we have been following, using the same hypotheses and tools? Based on what we know about the likelihood of confirming a cancer cluster and then identifying a cause looking only at environment -defined in the same way that it has generally been defined over the previous 40 years - without broadening our thinking, can we expect a different outcome when we look back 10 or 20 years from now? We suggest that the answer to these questions is “no” and that simply using the same approach, but with expending more resources will not get us closer to understanding cancer etiology. Certainly the results of this review indicate that despite the large number of geographic or community cancer cluster investigations and the amount of resources already expended, the likelihood of a successful cancer cluster investigation where the etiology of disease is discovered is extremely small. With four decades’ worth of cancer cluster investigations revealing little regarding cancer cluster etiology and prevention, it is time to recognize the shortcomings of the current approach to investigating cancer clusters investigations that originate with a perception of increased rates of cancer in a community and to begin a multidisciplinary national dialogue on more creative, innovative approaches towards understanding when and why cancer and other chronic diseases may cluster in time and space. In our view, the dialogue will need to include a focus on testable hypotheses based on well-defined measurable environmental exposures (e.g. concentrations of halogenated chemicals rather than “groundwater contamination”), specific disease outcomes (e.g. glioblastoma multiforme as opposed to “brain cancer”), methods for improving current and historical estimates of exposures and a broader examination of “environment” that would include biological, socioeconomic and lifestyle related factors.
